# Repair using the pectoralis major musculocutaneous flap for refractory anastomotic leakage after total esophagectomy

**DOI:** 10.1186/s40792-023-01659-y

**Published:** 2023-05-22

**Authors:** Yoko Oga, Tomoyuki Okumura, Takeshi Miwa, Yoshihisa Numata, Shigeki Matsumoto, Koji Kaneda, Nana Kimura, Mina Fukasawa, Masakazu Nagamori, Kosuke Mori, Naoya Takeda, Kenta Yagi, Miki Ito, Yasuhiro Nagaoka, Chitaru Takeshita, Toru Watanabe, Katsuhisa Hirano, Takamichi Igarashi, Haruyoshi Tanaka, Isaya Hashimoto, Kazuto Shibuya, Shozo Hojo, Isaku Yoshioka, Hideharu Abe, Toshihiko Satake, Tsutomu Fujii

**Affiliations:** 1 Department of Surgery and Science, Faculty of Medicine, Academic Assembly, University of Toyama, 2630 Sugitani, Toyama City, 930-0194 Japan; 2 Department of Otorhinolaryngology, Head & Neck Surgery, Faculty of Medicine, Academic Assembly, University of Toyama, Toyama City, Japan; 3 Department of Plastic, Reconstructive and Aesthetic Surgery, Toyama University Hospital, Toyama City, Japan

**Keywords:** Esophagectomy, Refractory anastomotic fistula, Pectoralis major musculocutaneous flap

## Abstract

**Background:**

The pectoralis major musculocutaneous flap (PMMF) is a pedicled flap often used as a reconstruction option in head and neck surgery, especially in cases with poor wound healing. However, applying PMMF after esophageal surgery is uncommon. We report here, the case of a successfully repaired refractory anastomotic fistula (RF) after total esophagectomy, by PMMF.

**Case presentation:**

A 73-year-old man had a history of hypopharyngolaryngectomy, cervical esophagectomy, and reconstruction using a free jejunal graft for hypopharyngeal carcinosarcoma at the age of 54. He also received conservative treatment for pharyngo-jejunal anastomotic leakage (AL), then postoperative radiation therapy. This time, he was diagnosed with carcinosarcoma in the upper thoracic esophagus; cT3rN0M0, cStageII, according to the Japanese Classification of Esophageal Cancer 12th Edition. As a salvage surgery, thoracoscopic total resection of the esophageal remnant and reconstruction using gastric tube via posterior mediastinal route was performed. The distal side of the jejunal graft was cut and re-anastomosed with the top of the gastric tube. An AL was observed on the 6th postoperative day (POD), and after 2 months of conservative treatment was then diagnosed as RF. The 3/4 circumference of the anterior wall of the gastric tube was ruptured for 6 cm in length, and surgical repair using PMMF was performed on POD71. The edge of the defect was exposed and the PMMF (10 × 5 cm) fed by thoracoacromial vessels was prepared. Then, the skin of the flap and the wedge of the leakage were hand sutured via double layers with the skin of the flap facing the intestinal lumen. Although a minor AL was observed on POD19, it healed with conservative treatment. No complications, such as stenosis, reflux, re-leakage, were observed over 3 years of postoperative follow-up.

**Conclusions:**

The PMMF is a useful option for repairing intractable AL after esophagectomy, especially in cases with large defect, as well as difficulties for microvascular anastomosis due to previous operation, radiation, or wound inflammation.

## Background

Anastomotic leakage (AL) after esophagectomy reportedly occurs in 3.0–30.0% of patients with an associated mortality rate of 7.2–35% [1, 2]. Although many ALs are treatable by conservative treatment, a small number of cervical ALs develop refractory anastomotic fistula (RF), a condition requiring surgical reconstruction [3]. The pectoralis major musculocutaneous flap (PMMF) is a well-established reconstruction technique for pharyngocutaneous fistula after pharyngolaryngectomy [4], but there are few reports of its use in treating RF after esophagectomy. We report a case in which the successful treatment of refractory gastric tube–cutaneous fistula after total esophagectomy was achieved by PMMF.

## Case presentation

A 73-year-old man presented to our hospital with the chief complaint of dysphagia. He had a history of hypopharyngolaryngectomy, cervical esophagectomy, reconstruction using a free jejunal graft, and permanent tracheostomy for hypopharyngeal carcinosarcoma at the age of 54 (19 years ago). In addition, he received conservative treatment for pharyngo-jejunal AL followed by radiation therapy. He also had a history of paroxysmal atrial fibrillation, right retinal artery occlusion, right internal carotid artery occlusion, cerebral artery stenosis, and hypothyroidism.

**Fig. 1:**
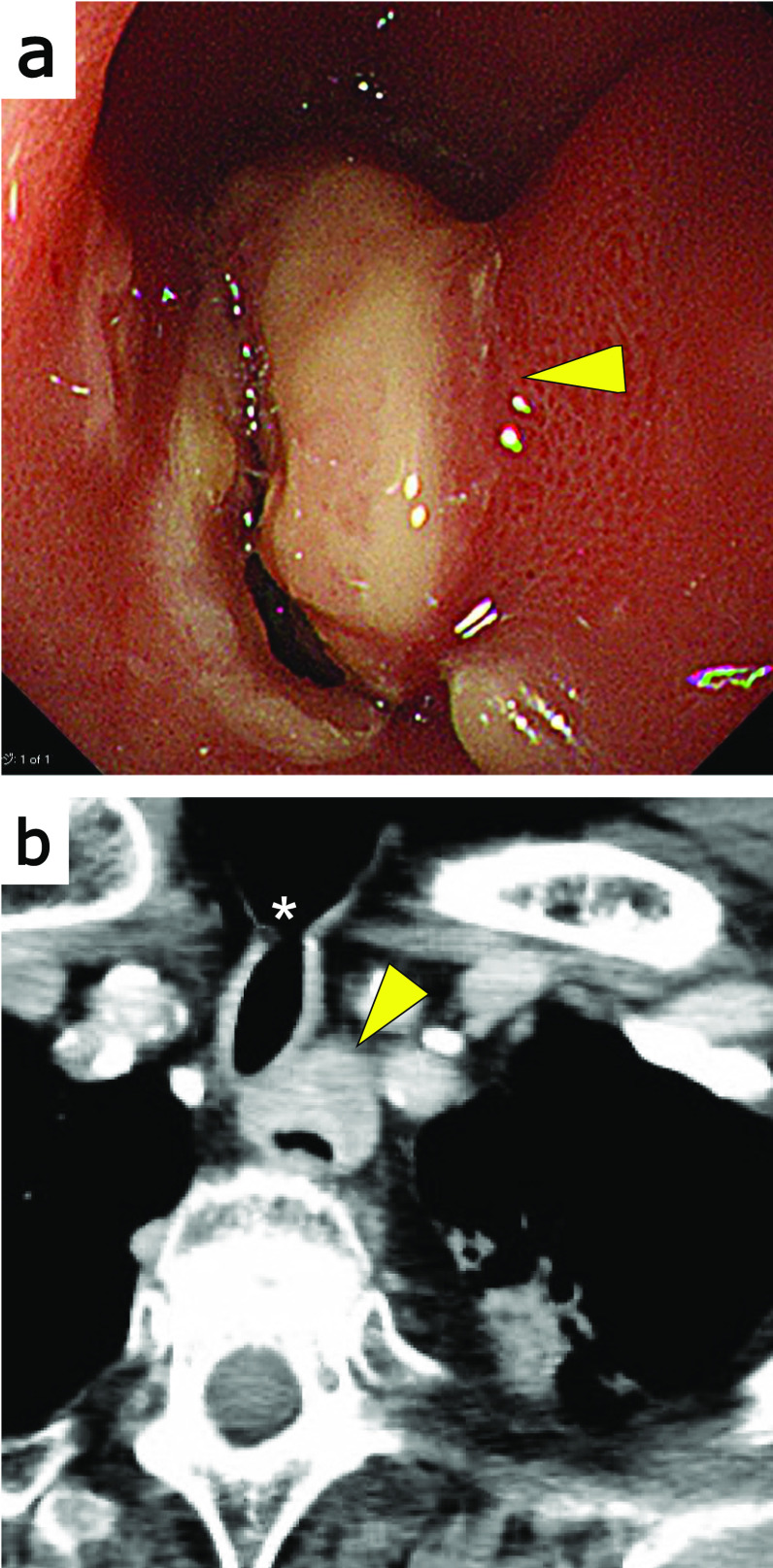
Preoperative findings. **a** Upper gastrointestinal endoscopy observed a tumor just below the esophago-jejunal anastomosis (yellow arrowhead). **b** Contrast-enhanced CT image showed the tumor at the esophageal side of the esophago-jejunal anastomosis (yellow arrowhead). Asterisk indicates tracheostomy

His upper gastrointestinal endoscopy revealed a mass lesion with submucosal elevation at the upper thoracic esophagus, below the esophago-jejunal anastomosis (Fig. 1a). Contrast-enhanced computed tomography (CT) showed an irregular wall thickening at the esophageal side of the esophago-jejunal anastomosis with esophageal stenosis (Fig. 1b). No lymph node or distant metastasis was detected. The pathologic diagnosis of the endoscopic biopsy revealed malignant spindle cell tumor including components of squamous cell carcinoma. Although it could not be determined, the tumor was not considered a recurrence of hypopharyngeal carcinosarcoma because 10 years had passed since surgery for hypopharyngeal carcinosarcoma. In addition, this time the tumor located in the thoracic esophagus, whereas in the previous surgery, the hypopharynx and cervical esophagus were removed with a free jejunum in between. Therefore, he was diagnosed with a carcinosarcoma of the esophagus; Ut, entire circumference, 40 mm, type1, cT3r N0 M0 cStage, according to Japanese Classification of Esophageal Cancer 12th Edition.

**Fig. 2:**
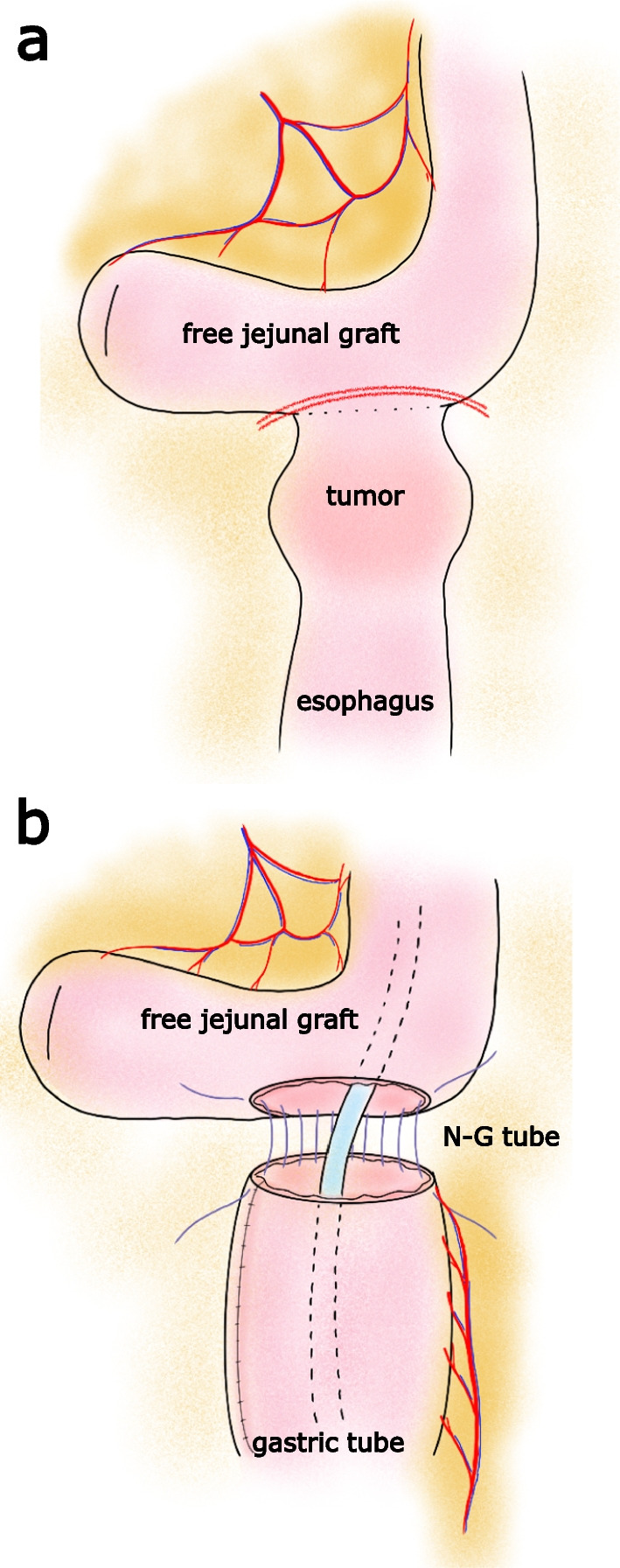
Operative findings of the total resection of the esophageal remnant. **a** The distal side of the jejunal graft was cut at 1 cm proximal to the esophago-jejunal anastomosis with preservation of the free jejunal graft. Double red line indicates the cutting line. **b** The cut end of the free jejunal graft was re-anastomosed with the top of the gastric tube which was pulled up via the posterior mediastinal route

Then thoracoscopic total resection of the remaining esophagus followed by reconstruction using a gastric tube via the posterior mediastinal route was performed. In the cervical procedure, due to significant existing scar-tissue and adhesions due to previous surgeries and radiation therapy, careful dissection was required to avoid injury of the free jejunal pedicle. The distal side of the jejunal graft was cut at 1 cm proximal to the previous anastomosis (Fig. 2a) and re-anastomosed with the top of the pulled-up gastric tube by hand suture (Fig. 2b).

**Fig. 3:**
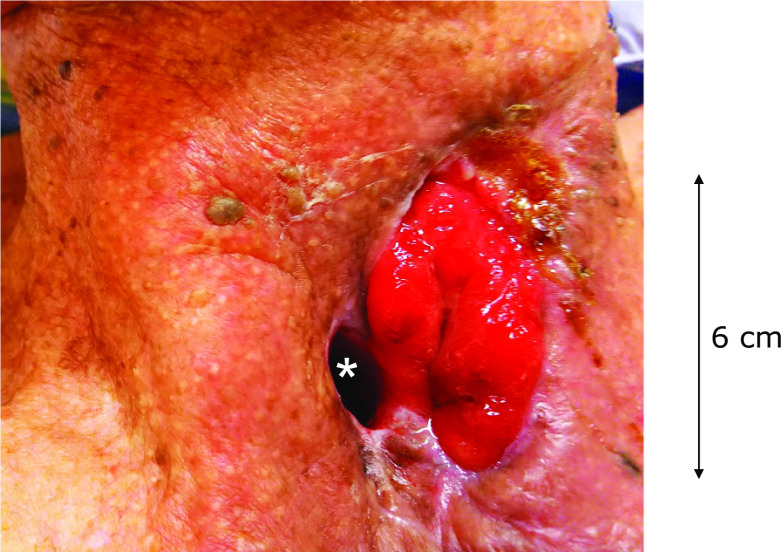
Refractory gastric tube–cutaneous fistula at POD62. The 3/4 circumference of the anterior wall of the gastric tube was defective. Asterisk indicates tracheostomy

A gastric tube–free jejunal anastomotic leakage was observed on the 6th postoperative day (POD6) with open drainage and daily wound irrigation continued. Although inflammation of the wound reduced after conservative treatment, the 3/4 circumference of the anterior wall of the gastric tube was ruptured for 6 cm in length (Fig. 3) and he was diagnosed as having a refractory gastric tube–cutaneous fistula that further conservative treatment was unlikely to heal.

**Fig. 4:**
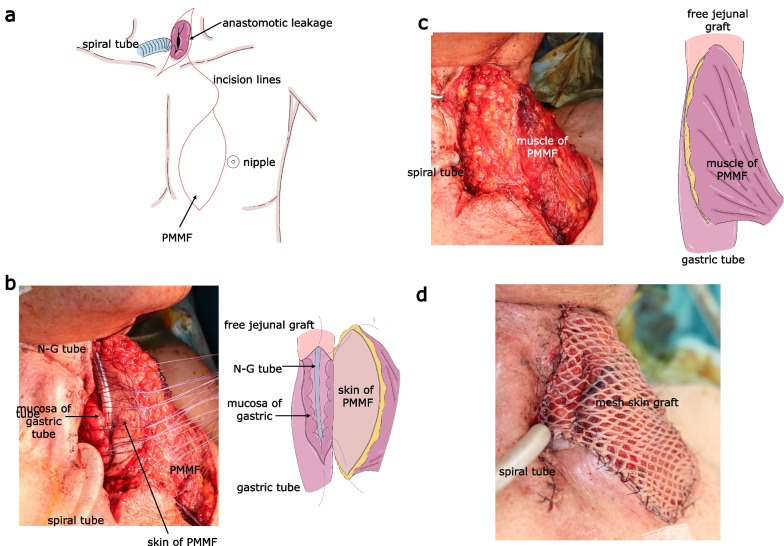
The operative findings of treatment using PMMF. **a** The skin incision was designed connecting the line for neck procedure and harvesting PMMF. **b** The skin of the PMMF and the wedge of the leakage were hand sutured via double layers with the skin inside. Scheme of the operative view is on the right. **c** The large defect of the anterior wall of the gastric tube was fully covered and repaired with PMMF. Scheme of the operative view is located on the right side. **d** The muscle side of the PMMF was covered with the mesh skin graft harvested from his left lateral thigh

On POD71, surgical repair of the refractory fistula was performed. The skin incision was designed, connecting the line for trimming the skin around the fistula in the neck and the line for harvesting PMMF in the left anterior thoracic region (Fig. 4a).

The edge of the leakage was exposed by carefully peeling away the surrounding tissue. Then the PMMF (10 × 5 cm) fed by thoracoacromial vessels and 4th anterior intercostal branches of the internal thoracic vessels, were prepared by integrating with skin, fat tissue and feeding vessels. Intraoperative blood flow visualization using indocyanine green (ICG) fluorescence confirmed sufficient blood supply at the edge of the anastomotic sites. Then the skin of the flap and the wedge of the leakage were hand sutured via double layers with the skin of the flap facing the intestinal lumen (Fig. 4b, c). The thick fat tissue of the PMMF was in contact with the outer wall of the permanent tracheostomy, not the suture line between the gastric tube and the PMMF. In addition, the omentum of the gastric tube could not be used due to scarring. Therefore, no additional tissue was placed to protect the permanent tracheostomy.

The muscle side of the flap was covered with a mesh skin graft harvested from his left lateral thigh (Fig. 4d). The operation time was 6 h and 27 min, with a blood loss of 335 mL.

**Fig. 5:**
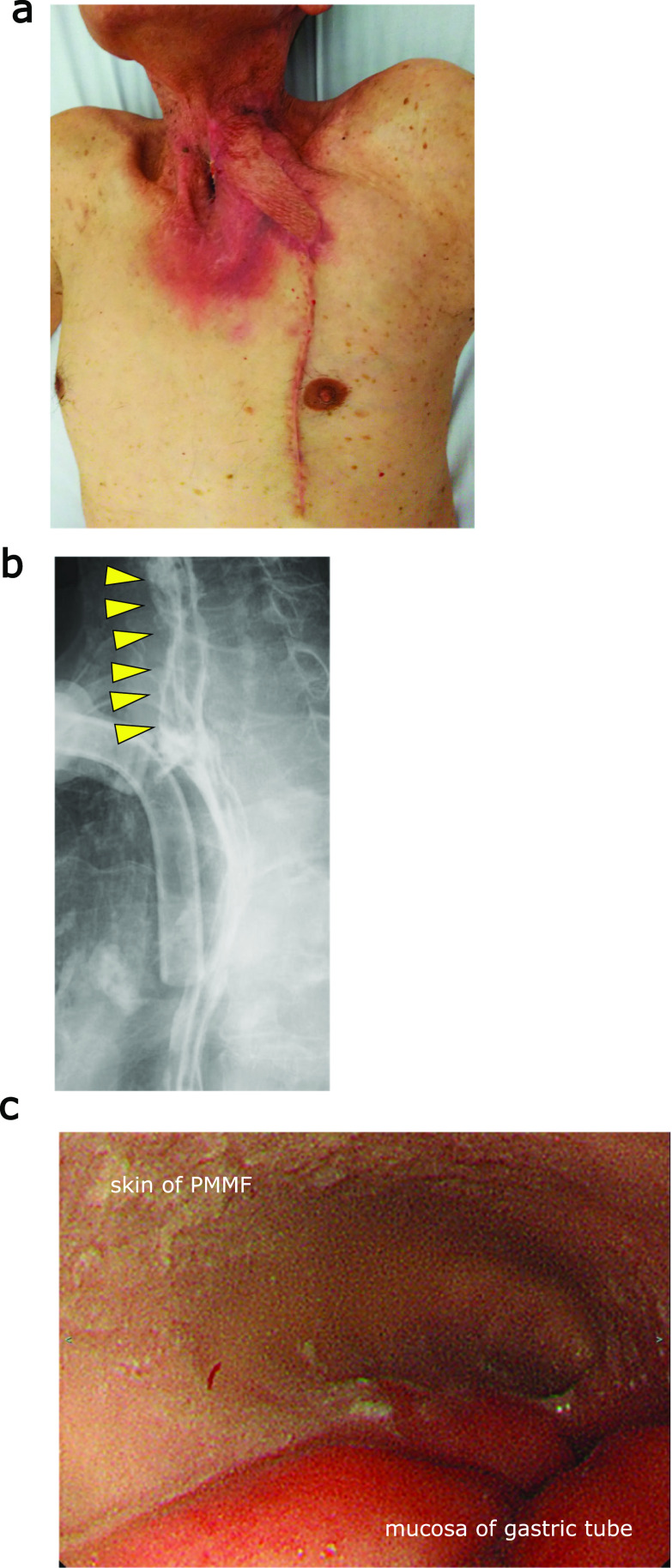
The postoperative findings of treatment using PMMF. **a** The appearance of the wound at POD 30. **b** Postoperative fluoroscopy at POD 33. The yellow arrowheads indicate the repaired site covered with PMMF. **c** Postoperative upper gastrointestinal endoscopy at 7 months after repair

Although a minor anastomotic leakage was observed on POD19, it healed with conservative treatment (Fig. 5a). Postoperative fluoroscopy showed a smooth passage at the repaired site (Fig. 5b) with oral intake started on POD33. He was discharged with good physical condition on POD60. No complications, such as ischemia or peptic injury of the skin, stenosis, or reflux, were observed (Fig. 5c) over 3 years of postoperative follow-up.

## Discussion

Many risk factors are associated with AL after esophagectomy, such as radiotherapy, diabetes mellitus, body mass index, age, congestive heart failure, atherosclerosis, smoking, that all exhibit insufficient blood supply at the anastomotic site [5–7]. In the present case, although the patient has several AL risk factors, a total esophagectomy was performed as it was the only curative treatment against the carcinosarcoma.

Most post-esophagectomy AL is successfully treated by conservative treatments, such as wound debridement, irrigation, and drainage [3]. However, a small number of ALs develop RF, defined as a non-curative anastomotic site–cutaneous fistula existing for longer than 2 months under conservative treatment [5].

In the present case, after debridement, irrigation, and antibiotic administration, the inflammation of the wound gradually healed, accompanied by expansion of the anastomotic wound defect. It takes about 2 months for the wound to stabilize without further changes, resulting in salvage surgery performed at POD71 (65 days after the onset of AL).

Surgical repair for RF after esophagectomy requires sufficient debridement of surrounding tissues, dead cavity filling, and patches larger than the leakage to prevent re-leakage and/or stricture. Autogenous tissues are used for the surgical repair of intractable AL. The free gastrointestinal graft, such as free jejunal graft, is favored for head and neck reconstructions, as the risks of stenosis or fistula are lower. However, this requires microvascular anastomosis [4]. A free flap, such as the anterolateral thigh flap, secures sufficient tissue volume, but also requires microvascular anastomosis [4]. On the other hand, a pedicle flap such as the PMMF, provides sufficient tissue volume yet doesn’t require microvascular anastomosis. Thus, it is used in situations where the free graft is not ideal [4, 8]. The PMMF is a readily available source of vascularized tissue, easily harvested for use in the head and neck. Especially in cases with poor wound healing, such as irradiated patients and those with postoperative salivary contamination, as the vascularized soft tissue coverage of this muscle flap is effective in preserving the major vessels [9]. Consequently, the use of a pedicled flap is broadly accepted as a reconstruction option in head and neck surgery [10]. On the other hand, reconstruction using PMMF after esophageal surgery is uncommon, accounting about 1% of AL reconstructions [4].

To date, 20 cases in total are reported have undergone reconstruction using PMMF for AL after esophagectomy (Table 1). Most of them were performed for RFs of esophago-gastric or esophago-colon anastomosis, at an average 68.5 days after initial surgery [4, 5, 8, 11–15]. In RFs following AL of anastomoses reconstructed by the subcutaneous route, the leakage defect was primary closed and then the PMMF was covered as reinforcement of the closure with the skin surface facing outside [8, 15].

**Table 1:** Literature review of repair using PMMF for AL after esophagectomy

Year of publication	Author	Ref. no.	Age	Sex	Reconstruction	Reconstruction route	Site of leakage	Size of defect	Side of skin	Repair after esophagectomy (days)	AL after repair
1998	Heitmiller	[14]	73	M	Stomach	Retrosternal	Esophago-gastric	1/2 circumference	Inside	ND	Present
1998	Williams JK	[11]	27	M	Colon	Retrosternal	Esophago-colon	Half of Anterior wall	Inside	11	Present
2006	Hirano M	[15]	61	F	Jejunal graft	Subcutaneous	Pharyngo-jejunum	3 cm	Outside	46	Absent
2010	McLean JN	[4]	ND	ND	ND	ND	ND	ND	Inside	ND	ND
2010	Morita M	[8]	55	M	Stomach	Subcutaneous	Esophago-gastric	ND	Outside	59	Absent
			54	M	Stomach	Subcutaneous	Esophago-gastric	ND	Outside	32	Absent
			90	M	Colon	Subcutaneous	Esophago-colon	ND	Outside	47	Absent
			70	M	Colon	Subcutaneous	Esophago-colon	ND	Outside	50	Absent
			66	M	Colon	Subcutaneous	Esophago-colon	ND	Outside	55	Absent
			61	M	Colon	Subcutaneous	Esophago-colon	ND	Outside	56	Present
2015	Yin K	[12]	46	F	Colon	Retrosternal	Esophago-colon	7 × 3 cm	Inside	ND	Present
2017	Yamana I	[5]	74	M	Stomach	Subcutaneous	Esophago-gastric	ND	ND	ND	ND
			69	F	Stomach	Subcutaneous	Esophago-gastric	ND	ND	ND	ND
			67	M	Stomach	Subcutaneous	Esophago-gastric	ND	ND	ND	ND
2020	Deng L	[13]	47	M	Stomach	Posterior mediastinal	Esophago-gastric	7 × 5 cm*	Inside	60	Absent
			68	M	Stomach	Retrosternal	Esophago-gastric	8 cm	Inside	60	Present
			56	M	Stomach	Posterior mediastinal	Esophago-gastric	7 × 5 cm*	Inside	240	Absent
			51	F	stomach	Posterior mediastinal	Esophago-gastric	7 × 5 cm*	Inside	60	Absent
			60	M	Stomach	Posterior mediastinal	Esophago-gastric	7 × 5 cm*	Inside	90	Absent
			49	F	Stomach	Posterior mediastinal	Esophago-gastric	7 × 5 cm*	Inside	90	Absent
2023	Oga Y	Present case	73	M	Stomach	Posterior mediastinal	Jejunal-gastric	6 cm	Inside	71	Present

*ND* not described, *AL* anastomotic leakage

^*^Size of the skin paddle to repair the defect

In RFs following retrosternal or posterior mediastinal reconstruction in which the defect was too large to primary close, PMMF was inserted to the defect with the skin side of the flap placed on the lumen side, avoiding both overextension and stenosis [11–14].

The skin directed toward the lumen was thought to prevent damage of the flap from exposure to digestive fluids, as well [11]. Re-leakage after repair was seen in some cases, but all have healed by conservative treatment, conceivably owing to the volume of the PMMF to fill the tissue defect [8, 11, 13, 14]. Reports have also shown that majority of the patients were able to start oral intake 10–15 days after PMMF repair, unless re-leakage was observed [8, 13, 15], providing us with a clinical indicator of when to start oral intake after surgery.

In the present case, the defect extended to 3/4 circumference of the anastomosis and was contaminated with digestive fluid, so primary closure was difficult. In addition, due to the previous reconstruction using a free jejunal graft and radiation therapy, as well as history of carotid artery occlusion, securing a recipient vascular bed for microvascular anastomosis was also considered difficult. Taken together, the PMMF was selected for reconstruction with the skin surface inward to successfully repair.

In post-irradiation cases such as the present case, considerable technical tips for designing the PMMF are to obtain a larger PMMF with sufficient thickness of fat tissue so that the gap could be filled after removal of scar tissue around the fistula, and to confirm the maintenance of blood flow at the margins with intraoperative blood flow visualization using ICG fluorescence.

## Conclusions

PMMF is a useful method to treat intractable anastomotic leakage after esophagectomy, especially in cases with large defect, as well as difficulties in microvascular anastomosis due to previous operation, radiation, and wound inflammation. This surgical option may enable surgeons to aggressively perform radical surgery for high-risk cases in which surgical resection is the only curative treatment.

## Data Availability

The datasets of this case report are available from the corresponding author upon reasonable request.

## Abbreviations

PMMF: Pectoralis major musculocutaneous flap
RF: Refractory anastomotic fistula
AL: Anastomotic leakage
POD: Postoperative day
CT: Computed tomography
